# Farm Typologies of Banana and Plantain Smallholders: Agricultural Practices and Disease Constraints in Department of Huila, Colombia

**DOI:** 10.1155/sci5/3357641

**Published:** 2025-12-30

**Authors:** Paula Bermeo-Fúquene, Edgar Mauricio Rico-Sierra, Edinson Bayardo Parra-Alferes, Diego Alberto Navarro-Niño, Angela Maria Vargas-Berdugo, Edgar Herney Varón-Devia, Eleonora Rodríguez-Polanco

**Affiliations:** ^1^ Department of Agriculture, Nataima Research Center, Corporación Colombiana de Investigación Agropecuaria (AGROSAVIA), Espinal, Tolima, Colombia

**Keywords:** crop suitability, elephantiasis, good agricultural practices, *Musa* spp., Sigatoka

## Abstract

Bananas and plantains (*Musa* spp.) are among the most widely cultivated crops in tropical regions and are consumed worldwide. These agricultural systems provide fundamental products in the Colombian basket of goods and generate the highest domestic rural employment. However, multiple crop types and diverse technologies implemented in *Musa* have not yet been characterized. This study aimed to classify smallholder *Musa* crop systems in one of the most productive *Musa* Department (Huila) and assess their relationships with the existing coffee system in this region. Seventy‐four farmers were interviewed in six municipalities of Huila, Colombia. The questionnaire‐based interview included four aspects related to producers: socioeconomic, locality, crop establishment, and crop management. Additionally, land use for *Musa* and coffee was assessed geographically. Based on producer answers, descriptive, proportional flow diagrams, factor analysis of mixed data (FAMD), hierarchical clustering on principal components (HCPC), and correlation analyses were performed. Most participants interviewed were male (91%, *n* = 67), had an elementary or high school education level (91%, *n* = 67), and were over 29 years old (96%, *n* = 71). Four groups of *Musa* crop systems were identified in four locations based on the implementation of Good Agricultural Practices and the main disease registered: (i) no Good Agricultural Practices certification (GAPc) in the southern subregion; (ii) GAPc in the center subregion; these two items show elephantiasis as the main disease; (iii) no GAPc in the center subregion; and (iv) GAPc in the northeastern subregion; these two clusters register Sigatoka as the main disease. All banana system localizations and 87.9% of plantain systems are highly suitable for coffee production. These findings support local government plans and *Musa* farm decision‐making aimed at increasing *Musa* production in Huila, Colombia.

## 1. Introduction

Bananas and plantains (*Musa* spp., hereafter *Musa*) are members of the Musaceae family and are among the most extensively cultivated (approximately 6 and 7 million ha, respectively) and consumed fruits worldwide [1, 2]. They grow in tropical and subtropical regions in more than 135 countries [3]. Unlike sweet bananas (*Musa* × *paradisiaca* AAB genome group) [4] that are commonly consumed as fresh fruit, plantains (*Musa* × *acuminata* AAA genome group) [4] are usually cooked before they are eaten, either boiled, fried, or roasted [5, 6]. In addition to these uses, in polyculture, *Musa* serve as shade plants for other crops such as coffee [7, 8]. These crops are crucial in most developing nations [9], being a fundamental product in the basket of goods, aiding in the caloric intake of subsistence farmers with limited incomes [10], and are an agricultural system that reduces poverty in rural communities [11]. Besides, *Musa* are among the leading agricultural systems that generate the highest rural employment, as is the case in Colombia [12].

In 2022, an estimated 485.000 ha of *Musa* were cultivated in Colombia [13], demonstrating its importance as a food and nutritional security asset for the nation. The Colombian southwestern region, particularly the Department of Huila, emerges as a key player, ranking in third position nationally with over 30,000 ha dedicated to plantain production [12], and accounting for 21.3% of the total agricultural area in the Department [14]. *Musa* research and industrialization [15, 16] have increased in Huila due to local government investment [17, 18]. More than 500 families in Huila rely on *Musa* crops for their livelihoods [17], and these crops contribute to rural employment for more than 960,000 people through Colombia [14]. However, there are still no official data on the actual extent of *Musa* crop producers in the Huila region, limiting the development of evidence‐based strategies for governmental management and sectoral improvement.

Farm characterization (typology) provides an essential framework for understanding agricultural systems based on agronomic, economic, marketing, and social characteristics. These features are often heterogeneous across farms, grouping them into distinct categories [19, 20]. Typology enables the development of targeted interventions and policies [21–23], in Germany [24] and Kenya [21], as case studies. In developing countries, typification establishes differential strategies that enhance productive conditions with adequate management recommendations [25–27], creating context‐specific solutions and contributing to food security in dairy [28], passion fruit [29], and backyards [30]. Therefore, typology can ensure that stakeholders’ interventions are suitable for the different circumstances of farmers in emerging economies [31]. In Cameroon, for instance, forty growers were interviewed, and their differentiation was based on the vegetation structure, management practices, and plantation age [32]. Brazilian dairy farms were typified according to economic and production characteristics [33]. In the case of plantain, Abiola et al. [34] demonstrated the existence of three types of farms in the Republic of Benin, mainly differentiated by the type of crop system, i.e., organic dryland, backyard traditional gardens, and intensive cropping systems.

In developing nations, sustainable agricultural practices are crucial for ensuring food security and enhancing rural livelihoods. Polyculture, primarily through intercropping, has emerged as an essential agricultural strategy in these regions [35–37]. By cultivating multiple crops simultaneously in the same area, polyculture not only maximizes land use but also increases resilience to pests [38], diseases [39], and climatic uncertainties [40, 41]. This practice boosts productivity, reduces risks, and supports biodiversity, making it an essential approach for sustainable agriculture in developing economies.

In Colombia, crop characterization has been tested in coffee [42], citrus [43], cacao [44, 45], and passion fruit [29]. In the case of *Musa*, producers are classified according to the crop area: small (0.1–5 ha), medium (5.1–15 ha), large (15.1–30 ha), and industrial (> 30.1 ha) [46]. Another way to classify Musa producers is by productivity: high, medium, medium‐low, or low productivity [47]. However, research on Musa farm typification in the Huila Department has not been conducted. *Musa* crops are vital for rural economies in Colombia, especially in the southwest region, where they contribute to food security and employment [12, 45]. Therefore, farm characterization in the Huila Department must offer a comprehensive understanding of farming systems, including their interactions with other crop systems, to identify key factors influencing productivity, such as technology use and management practices. By classifying farms based on their practices, local and national governments can develop effective policies and initiatives that support prospective producers and address the challenges faced by low‐productivity farms.

No prior research has evaluated the Musa farm typification in the Huila Department (Colombia). Accordingly, this study aimed to determine which socioeconomic (i.e., demographic conditions: income, education) and agronomic variables (i.e., phytosanitary conditions, use of pesticides) best explain the differentiation of *Musa* crop producers in the Huila region of Colombia and their interactions with coffee crops. Because the use of social characteristics has not been important in the differentiation of typology in several studies [32–34], this research hypothesizes that *Musa* farm characterization in Huila will depend mainly on agronomic variables and crop characteristics (pest predominancy, age crop) rather than socioeconomic (demographic) ones. This study will provide a better understanding of the relationships between *Musa* crop systems and productivity in the Colombian context as a food security asset for the nation. The results could significantly enhance crop management strategies and boost farm‐level productivity, improving regional food security and creating more equitable opportunities for rural communities to thrive.

## 2. Materials and Methods

### 2.1. Study Area

**Figure 1:**
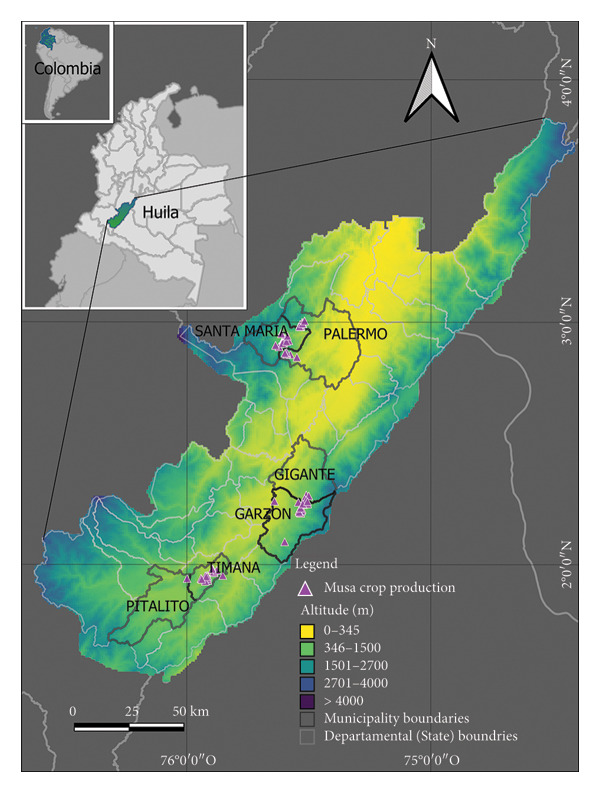
Smallholder Musa crop production system (magenta triangles) location map in the southwestern region of Colombia showing the municipalities (lower administrative unit) and subregions included. The municipalities of Santa Maria and Palermo are located in the northern subregion, while the municipalities of Gigante and Garzón are found in the center subregion. The municipalities of Pitalito and Timaná are located in the southern subregion. Altitude was used as the base map.

This study was conducted in 2021 across the three main subregions and six municipalities (lower administrative units) of the Department of Huila, including Garzón, Gigante, Pitalito, Santa María, and Timaná, in Colombia. This Department (State) is located in the southwest of the Andean region (Figure 1). Annual precipitation and mean temperature vary according to the municipality, ranging between 690 mm and 1320 mm and 20.5°C and 27°C ([48]; Table 1), with a tropical climate (*A* *w*) according to the classification of Köppen [52]. Since there was only one crop system in Pitalito, this farm was included in the Timaná group for data analysis.

**Table 1:** Climatic characteristics of the subregions in Huila, Colombia, based on official weather stations and governmental information.

Subregion	Municipality	Elevation (town center) (m a.s.l.)	Annual rainfall (mm)	Mean temperature (°C)	Source
Northern	Palermo	690	1755.2	27	Alcaldía de Palermo [49]
	Santa María	1320	1589.6	24	

Center	Garzón	828	1246.9	20.5	IDEAM [48]
	Gigante	809	1165.9	24	

Southern	Pitalito	1318	1550.7	20.5	IDEAM [48]; Fick and Hijmans [50]
	Timaná	110	1528^∗^	24	

^∗^The data were calculated using terra package [51] from WorldClim2 data.

### 2.2. Selection of Farm Households and Data Collection

Three potential subregions in the Huila Department (State), due to their importance for the local government, were selected to characterize the Musa production systems and their relationship with the socioeconomic status of producers [53, 54]. A semistructured questionnaire was designed based on different aspects, according to Rodriguez‐Polanco et al. [29], to collect (i) socioeconomic, (ii) locality, (iii) crop establishment, and (iv) crop management information. Since no official information was available about the total number of Musa crop producers in the region, our sampling sufficiency was achieved when a nominee of the latest interviewee had already been interviewed in an area. Thirty‐five variables (three continuous and 32 categorical) were used for the statistical analysis (Table 2). Before starting the survey, crop producers were informed about the study project and the confidentiality of their information. The snowball sampling technique was employed to gather all the possible *Musa* producers in subregions (Figure 2(a)). In each semistructured interview, the interviewees suggested other potential participants [55, 56]. The snowball sampling is a nonprobabilistic method that aids researchers in selecting units that represent a population [57]. This technique is useful when accessing all target areas is difficult [58] and limited financial resources for a probabilistic survey exist [59], contrary to stratified and random sampling techniques [60]. In addition, the snowball technique has been accepted in diverse typification studies [25, 59, 61–63]. This approach enabled the identification of additional producers through referrals from initial participants, thereby the sample within each locality (Figure 2(a)) [64]. The snowball sampling method is inherently prone to selection bias, as the sample depends on the social connections of the initial respondents rather than random selection [58]. In this study, this technique was employed due to the absence of an official registry of *Musa* producers in the Huila Department. Nonetheless, to reduce bias from the sampling method, the interviewed ones, farmers, were instructed to avoid contacting the next recommended participant until all interviews were completed; the identity of the person who provided each next nomination was kept confidential [59]. The study was ethically approved by AGROSAVIA, and prior to the start of the survey, producers were informed of the study’s purpose. All producers participated voluntarily in this study and signed a document about their given information. Geographical coordinates were measured using a GPS to map the farms.

**Table 2:** The semistructured survey categorized the *Musa* crop systems in the southwestern region of Colombia, considering four aspects: (1) socioeconomic, (2) locality, (3) crop establishment, and (4) crop management.

Aspect	Variable (acronym)	Type of variable	Category (abbreviation)
(1) Socioeconomic	Age of producer	Categorical	< 29 years old
			29–59 years old
			≥ 60 years old

(1) Socioeconomic	Years of crop knowledge	Categorical	< 10 years
			10–30 years
			≥ 30 years

**(1) Socioeconomic**	**Bank credit**	**Categorical**	**Bank credit**
			**No bank credit (NoCreBank)**

(1) Socioeconomic	WhatsApp use	Categorical	WhatsApp use
			No WhatsApp use

(1) Socioeconomic	Technical training	Categorical	ASOHOFRUCOL training
			No technical training

(1) Socioeconomic	Educational status of the producer	Categorical	Elementary school
			High school
			Graduate/bachelor

(1) Socioeconomic	Gender of producer	Categorical	Female (F)
			Male (M)

**(1) Socioeconomic**	**Internet use**	**Categorical**	**Internet**
			**No internet**

(1) Socioeconomic	Sewer system	Categorical	Sewer system
			No sewer system

**(1) Socioeconomic**	**Water source**	**Categorical**	**Torrent**
			**Another water source (OtherWaterS)**

**(1) Socioeconomic**	**Workforce**	**Categorical**	**Only familiar (FamilyWork)**
			**Extra familiar (ExtraWork)**
			**Familiar and extra familiar (FamExtraWork)**

(2) Locality	Topography	Categorical	Shallow slope (0%–7%)
			Moderate slope (7%–25%)
			Steep slope (> 25%)

(3) Crop establishment	Polyculture Musa area	Continuous	NA

**(3) Crop establishment**	**Monoculture**	**Categorical**	**Monoculture**
			**No monoculture**

(3) Crop establishment	Crop association	Categorical	Coffee crops
			Coffee crops and trees
			No crops associated

(3) Crop establishment	Crop irrigation	Categorical	Crop irrigation
			No crop irrigation

**(3) Crop establishment**	**Musa area**	**Continuous**	**NA**

**(3) Crop establishment**	**Plant material**	**Categorical**	**Dominic Harton**
			**Other plant material (OtherPlantM)**

**(4) Crop management**	**Good Agricultural Practices certification (GAPc)**	**Categorical**	**Certified in GAP (GAP)**
			**Not certified in GAP (NoGAP)**

**(4) Crop management**	**Main disease**	**Categorical**	**Elephantiasis**
			**Sigatoka**
			**Other**

**(4) Crop management**	**Fruit quality**	**Continuous**	**NA**

**(4) Crop management**	**Soil analysis**	**Categorical**	**Soil analysis (SoilAn)**
			**No soil analysis (NoSoilAn)**

**(4) Crop management**	**Water analysis**	**Categorical**	**Water analysis (WaterAn)**
			**No water analysis (NoWaterAn)**

(4) Crop management	Wrap branch	Categorical	Wrap bunch (wrap)
			Without wrap bunch (NoWrap)

(4) Crop management	Yield	Continuous	NA

(4) Crop management	Pest management	Categorical	Chemical pest management
			Organic and cultural pest management
			No pest management

(4) Crop management	Pest management frequency	Categorical	1–6 months
			≥ 6 months
			No pest management

(4) Crop management	Disease management	Categorical	Cultural disease management
			Organic and chemical disease management
			No disease management

(4) Crop management	Weed management	Categorical	Mechanical weed management
			Mechanical and chemical weed management
			No weed management

(4) Crop management	Nutrition management	Categorical	Chemical nutrition management
			Chemical and organic nutrition management
			No chemical nutrition management

(4) Crop management	Plant pruning	Categorical	Plant pruning
			No plant pruning

(4) Crop management	Pseudostem elimination	Categorical	Pseudostem elimination
			No pseudostem elimination

*Note:* Rows in bold represent the variables used in the FAMD analysis.

Abbreviation: NA = not available.

Figure 2Total sample and variable selection of smallholder *Musa* crop production systems in Huila (Colombia). (a) Study flow diagram illustrating the process used to arrive at the final sample size of 74 Musaceae producers in the three subregions. The initial “seed” consisted of the first interviews of *Musa* crop producers conducted in each subregion, which enabled the identification of additional Musaceae producers in these areas. (b) Variable selection process for Musa crop system typification in Huila, Colombia.

**Figure:**
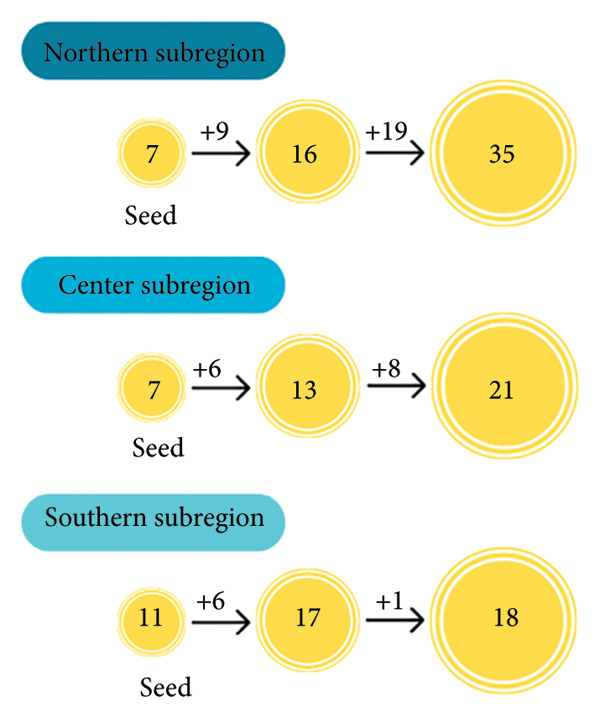
(a)

**Figure:**
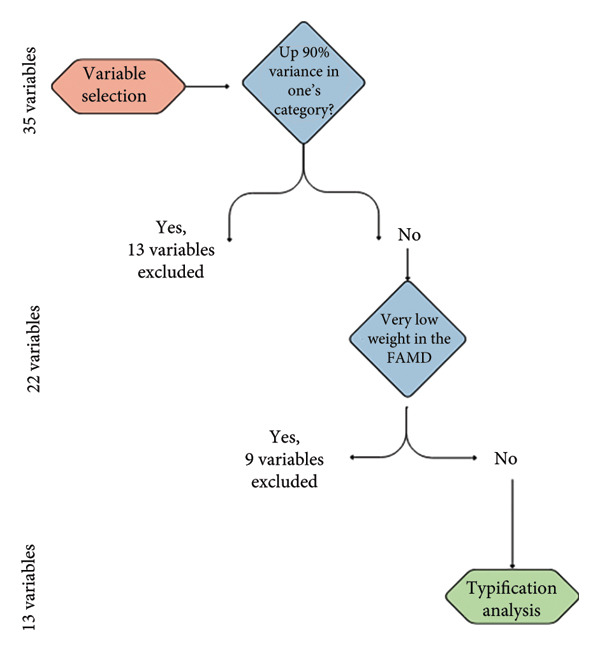
(b)

### 2.3. Farm Characterization and Statistical Analysis

A descriptive analysis was conducted to characterize *Musa* farms. A category that presented the distribution of equal to or higher than 90% per variable was exclusively considered for descriptive analysis, since such a variable may not contribute to the typification [65]. Thus, 91% of the participants were males; in this case, the variable gender was excluded from the factor analysis of mixed data (FAMD) analysis because there is a slight variation between *Musa* crop systems (Figure 2(b)). Later, to identify the main variables that define the typologies in each subregion, the FAMD was used with the variables. Due to the initial low accumulated variance, this method was repeated using the variables that retained 91.8% of the total variation (13 variables) (Figure 2(b)). The results from the FAMD were used to perform a hierarchical clustering on principle components (HCPC) analysis to create the *Musa* farm groups, using the chi‐q test to detect statistical differences between the clusters. All analyses were tested in the RStudio program [66], using the FactoMineR and factoextra packages.

The categorical variable age of the producer was determined according to individuals under 29 years old classified as the young population by the Law 1622 of 2013 of the Republic of Colombia [67]. Those aged 60 years or older are defined as older adults by the Law 2055 of 2020 [68]. Consequently, individuals between 29 and 59 years old are considered adults by exclusion of the two previously defined categories. The topography variable was assessed using Garmin® (GPSMAP 62 Series), based on the localization and topographic maps. Then, the criteria of shallow (0%–7%), moderate (7%–12%), and steep (> 12%) were considered according to IGAC [69]. Finally, the fruit quality was determined according to the percentage of first selection. The first selection is determined according to the Colombian Law [70], which indicates three *Musa* quality classifications. Thus, the percentage of fruit quality in this case was the proportion of first selection (kg)/total *Musa* production (kg) in a farm.

### 2.4. Correlation Analysis

A correlation analysis was conducted to understand whether there was a relationship between variables used in the characterization analysis, employing as a parameter the correlation coefficient *f*_*K*_ [71], a versatile and applicable statistical tool for comparing categorical and continuous variables. The analysis was conducted in Python, and a value over 0.7 was accepted as a strong correlation [72]; the significance was performed using a *Z* score at a 95% confidence level.

### 2.5. Land Use Analysis

Suitability maps available from the Agricultural Rural Planning Unit (UPRA, for its acronym in Spanish) portal [73, 74] were used to identify the direct spatial relationships between the Musa system and coffee‐growing areas in the Department of Huila, maintaining five suitability categories: high, medium, low, and zero suitability, and legal exclusion. The high suitability category includes areas exhibiting optimal conditions from a physical, socioecosystemic, and socioeconomic perspective; the medium suitability category contains areas characterized by moderate limitations, whether physical, socioecosystemic, or socioeconomic in nature; the low suitability category includes areas with significant physical, socioecosystemic, socioeconomic limitations, or all of these, which could potentially be adapted through substantial investments, the development of new technologies, or both; the no suitability category is composed of areas with physical and socioecosystemic constraints that render the development of the activity unfeasible; the legal exclusion category includes areas where, by legal mandate, the development of commercial crops is not possible [14, 74].

Occurrence data from *Musa* farms collected in the current study were classified based on the plant material in the Department of Huila. Then, the TMap interface [75] was used to visualize the relationship between crop suitability and farm location. For coffee, *Musa* system localizations were plotted. The suitability maps for coffee and plantain were created using the “vect” and “intersect” functions from the terra package [51]. All the suitability categories (low, medium, and high) were used to consider the intersection between coffee and plantain aptness in the Department of Huila. Finally, the function *summarize* from dplyr [76] was used to calculate the total area for each suitability category.

## 3. Results

### 3.1. *Musa* Crop System Characterization

Specific patterns in the Huila Department were identified in the descriptive analysis of the smallholder *Musa* crop systems. The socioeconomic aspect shows that 91% of the farmers were males, with only elementary or high school formation (90%), and 96% were older than 29 years. Technical assistance has been provided to 95% of producers, primarily by the Colombian Horticultural Association (ASOHOFRUCOL, for its Spanish acronym). Additionally, the primary training crop system topics focused on technical support in *Musa* production. Regarding crop status, 91% of *Musa* crop systems employ crop fertilization. However, according to the survey, these products are incorporated according to coffee nutrient requirements, and the quantity applied generally depends on the fertilization needs of the remaining coffee crop production. We have also found that 94.6% of *Musa* producers have coffee crops in their lands. In terms of sanitary conditions in the crop systems, despite the widespread incidence (100%) of the South American Palm Weevil (*Cosmopolites sordidus,* Coleoptera: Curculionidae), almost all *Musa* producers (99%) do not perform pest monitoring. The major diseases found were Sigatoka (50%) and banana elephantiasis disease (BED) (43%). The principal control for avoiding phytosanitary aboveground problems in *Musa* was leaf (92%) and pseudostem (93%) elimination, and 80% of the producers wrapped fruit bunches.

### 3.2. Farm Characterization

According to the farm typology derived from semistructured survey results (FAMD/HCPC analysis), four different groups were identified based on their localization (subregion) (Table S1). The first two groups correspond to the center and southern subregions in Huila, respectively. The Municipality of Timaná formed the first cluster (Group 1), where family workforce (94.4%), no bank loan for agricultural purposes (55.5%), and a low crop land surface (2 ± 1 ha) lead to minimal interest in cultivating *Musa* crops in this subregion (Table 3, Figure 2).

**Table 3:** *Musa* characterization in Huila, Colombia, according to the subregion: south (Timaná), center (Garzón and Gigante), northwest (Santa María), and northeast (Palermo).

Variable	Group 1 southern subregion: Timaná (*n* = 18)	Group 2 center subregion: Garzón and Gigante (*n* = 21)	Group 3 northwestern subregion: Santa María (*n* = 19)	Group 4 northeastern subregion: Palermo (*n* = 16)
Bank loan	No (55.5%)	Yes (80.9%)	Yes (89.5%)	Yes (87.5%)
Good agricultural practices certification	No (100%)	Yes (processing) (100%)	No (100%)	Yes (100%)
Internet	Yes (55.5%)	Yes (81%)	No (74%)	Yes (81.25%)
Main disease	Elephantiasis (100%)	Elephantiasis (57%)	Sigatoka (84.2%)	Sigatoka (100%)
Monoculture	No (100%)	No (90.5%)	No (78.9%)	Mono (50%) and polyculture (50%)
Musa area	2.0 ± 1.0 ha	1.5 ± 1.2 ha	2.76 ± 1.7 ha	3.18 ± 1.5 ha
Plant material	Gros Michel (banana) (94%)	Dominico Hartón (plantain) (100%)	Dominico Hartón (plantain) (100%)	Dominico Hartón (plantain) (100%)
First quality^∗^	57.2 ± 38.5%	88.1 ± 11.5%	94.2 ± 7.3%	81.75 ± 18.3%
Soil analysis	No (77.7%)	No (85.7%)	No (63.1%)	Yes (100%)
Water analysis	No (94.4%)	No (80.9%)	No (100%)	Yes (100%)
Water source	Countryside water supply (88.8%)	Stream (57.1%)	Stream (89.47%)	Torrent (56.25%)
Work force	Only familiar work (94.4%)	Familiar and external (52%)	Familiar and external (57.9%)	Familiar and external (100%)
Yield	1232.3 ± 1355.1 t·ha^−1^·year^−1^	1051.4 ± 625.1 t·ha^−1^·year^−1^	1111.8 ± 722.6 t·ha^−1^·year^−1^	1811.4 ± 1102.9 t·ha^−1^·year^−1^

*Note:* The variables per typology were considered when occurring at a frequency of > 51%. The “*n*” value in the column header represents the total number of farms. The percentage (%) per category is the frequency within each group. For quantitative variables, the mean ± standard deviation is shown, according to the HCPC analysis [77].

^∗^Fruit quality assessment represents the percentage (%) of the best fruit quality compared to other qualities (medium and low).

The second cluster (Group 2) mainly in the center subregion (Garzón and Gigante) was characterized by the implementation of Good Agricultural Practices certification (GAPc) in their plantain areas (all plant material is certified 100%). Contrary to the previous cluster, producers are interested in *Musa* crop investment, using extra workforce for agricultural activities (52%). Despite the interest in GAPc implementation, routine analysis of soil and water (85.7% and 80.9%, respectively) has not been carried out during production. BED is recognized as the principal disease in this cluster.

Groups 3 and 4 were geographically situated in the northern subregion (Table 3). Group 3 (Municipality of Santa María) was characterized exclusively by the Dominico Harton plant material, and the lack of GAPc likely contributed to Sigatoka being the primary disease (84.2%). BED only represents 10.5% of this cluster. Finally, Group 4, represented by the northeastern subregion in Huila (Palermo Municipality), displays the most substantial investment and economic importance in *Musa* production (Dominico Hartón plantain in this case) compared to other localities. Furthermore, GAPc implementation and routinary soil and water analysis were completed in the farms included in this cluster (100%).

Figure 3 illustrates the primary structural characteristics that distinguish farms across the four subregions in the Huila Department. The subregion GAPc–plant disease pathway displays that the northeastern and center subregions possess GAPc, whereas northwestern and south subregions predominates the lack of GAP‐certified farms (Figure 3(a)). In the case of the subregion–plant material pathway, the predominance is for Gros Michel in the southern subregion and Dominic Hartón in the center and northern area (Figure 3(b)). The subregion–workforce pathway evidences that family‐only labor is characteristic of the southern subregion, while mixed family and external labors predominate in the center and northern municipalities.

Figure 3
*Musa* farm characterization pathway for farms in southwestern Colombia regarding (a) subregion GAPc process and plant disease certification, (b) subregion–plant material, and (c) subregion–workforce. FamExtraWork = Familiar extra work.

**Figure:**
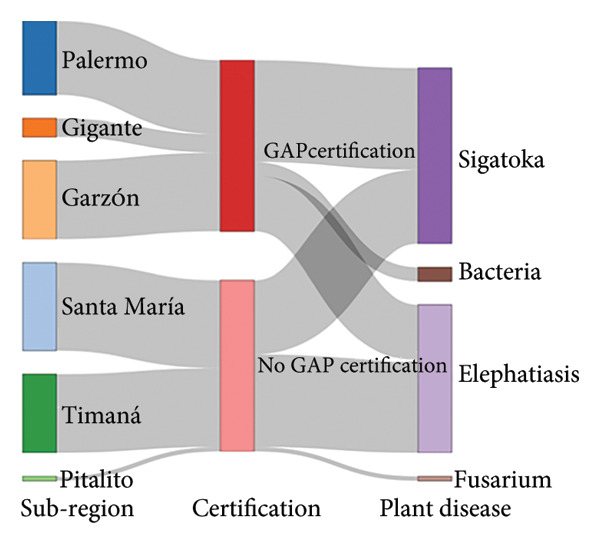
(a)

**Figure:**
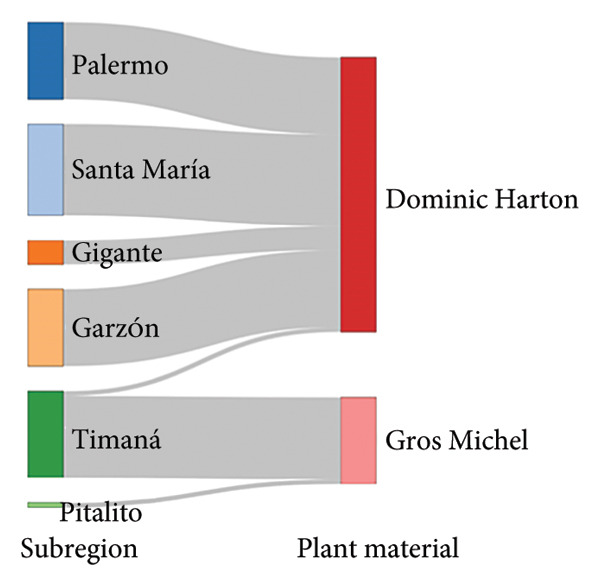
(b)

**Figure:**
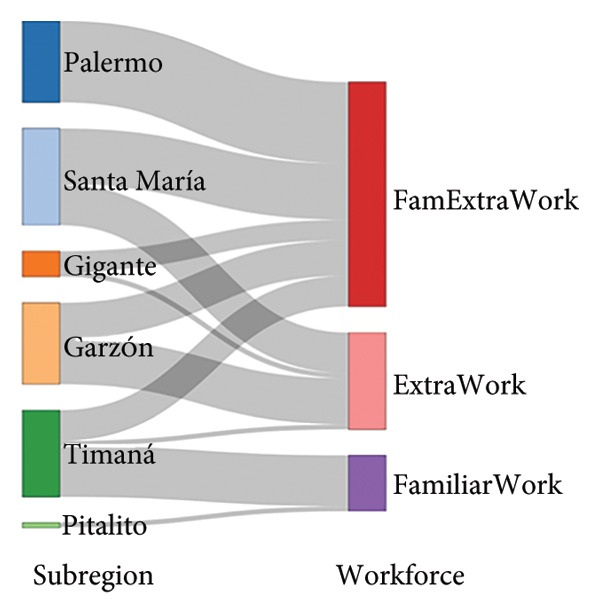
(c)

### 3.3. Correlation Analysis

**Figure 4:**
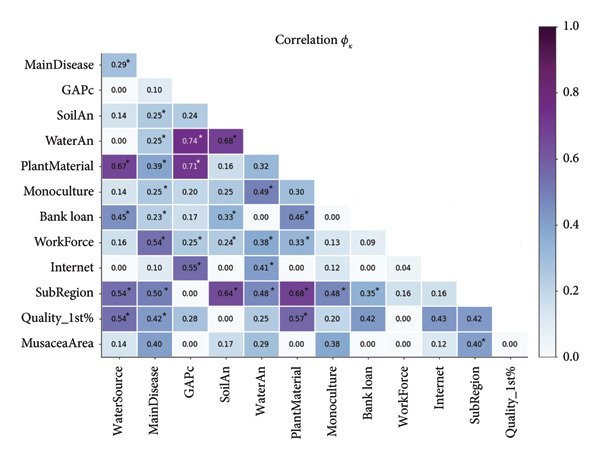
Correlation matrix (*f*_*k*_ index) between variables that define *Musa* producers in the Colombian Huila Department. ^∗^A statistically significant difference of 95% was calculated by the confidence interval according to the *Z* score. GAPc = Good Agricultural Practices certification, SoilAn = soil analysis, WaterAn = water analysis, Quality_1st% = percentage of high‐quality *Musa* production.

The correlation analysis shows that GAPc was strongly correlated with plant material and water analysis (0.71 and 0.74, respectively) (Figure 4). This result reflects the link between certification requirements and crop management practices, particularly in the center and northeastern subregions. No strong correlations were found between socioeconomic variables and crop management attributes, reinforcing the finding that typologies are mainly structured by management and phytosanitary factors rather than by socioeconomic characteristics.

### 3.4. Land Use Analysis

*Musa* and coffee crops have suitability variations across the Huila region (Figure 5). Both banana and plantain have a significant proportion of areas classified as not suitable, with 62.5% and 50.14%, respectively. In contrast, coffee has the lowest proportion of not suitable areas, with 34.4%. The total suitable area, i.e., high, medium, and low suitability in the coffee area, is 684,584 ha, representing 37.7% of the total area of Huila. Low suitability for the banana crop area represents 8.6% compared to the total Huila surface. Most banana farms surveyed are located in not suitable regions (Figure 5(a)). In the case of plantain, the south subregion in Huila has the largest high suitability area. However, it represents only 9.1% of the area in the Department. All banana farms are situated in areas of high suitability for coffee, and the majority of plantain farms also fall within suitable areas (Figure 5(c)). When considering the combination of coffee and plantain, the total suitable area amounts to 555,104 ha or 30.6% of the total Departmental (State) area.

Figure 5
*Musa* and coffee suitability areas in the Huila Department (Colombia). Suitability was evaluated for (a) banana, (b) plantain, (c) coffee, and (d) coffee and plantain (merged). High suitability (purple polygon): areas with optimal conditions from different perspectives; medium suitability (turquoise polygon): areas characterized by moderate limitations; low suitability (yellow polygon): areas with significant limitations, which could be adapted through substantial investments, the development of new technologies, or both; not suitable (white polygon): areas with several constraints; and legal exclusion (gray polygon): areas where, by legal mandate, commercial crop production is not allowed. The green polygon in (d) represents the intersection of the potential suitability for coffee and plantain. The banana system zooms 8 km; the plantain system zoom scale bar in the north and center is 15 km and 20 km, respectively.

**Figure:**
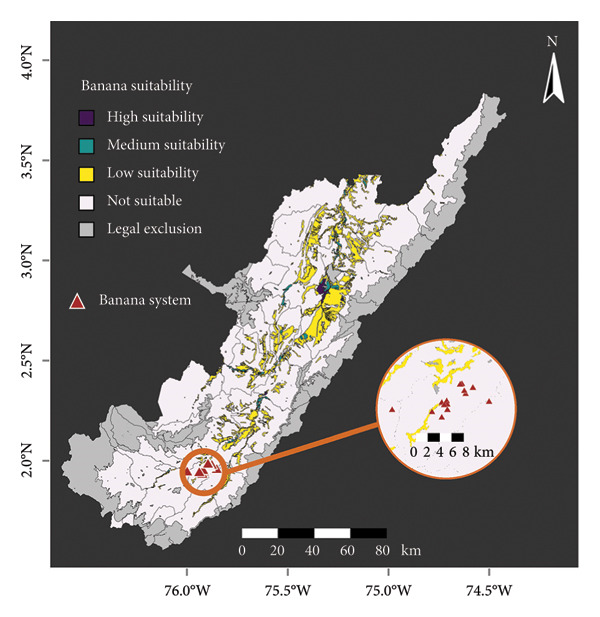
(a)

**Figure:**
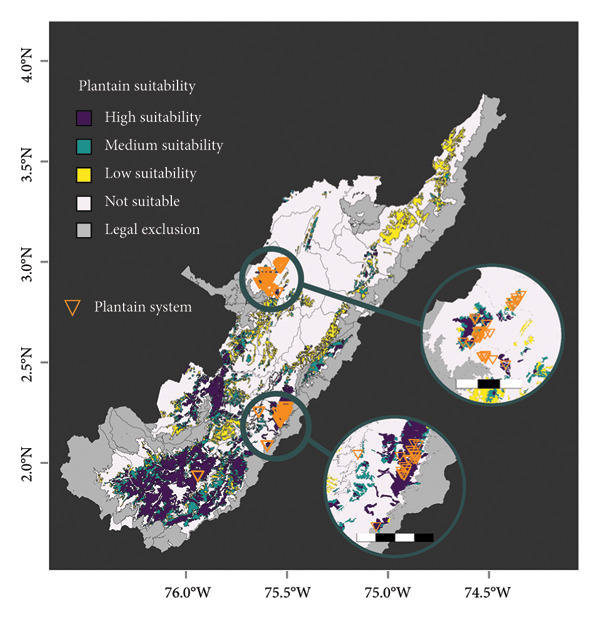
(b)

**Figure:**
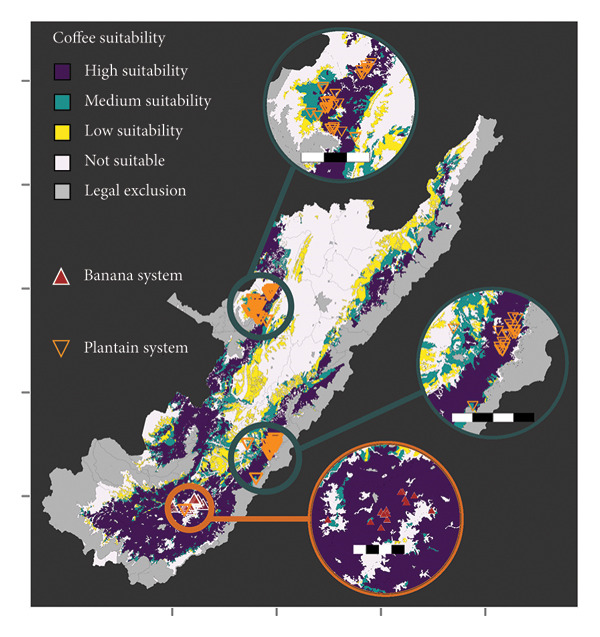
(c)

**Figure:**
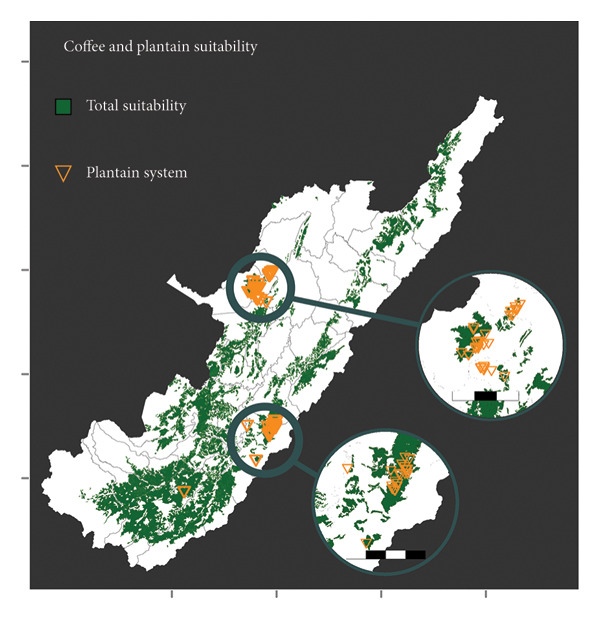
(d)

## 4. Discussion

This study provides a detailed account of the vital role local practices play in *Musa* (banana and plantain) production in the Huila Department of Colombia, as well as their connection to coffee crop systems. Results show that two *Musa* subregions (northeast and center) are associated with GAPc, whereas this aspect is not predominant in the south and northwestern farms. The prevalence of the devastating BED is noteworthy in the southern and center subregions, in contrast to the northern subregions where Sigatoka disease emerges as the primary phytosanitary concern. According to the land use area, plantain and coffee crops are suitable in the Huila Department. Traditionally, Colombian *Musa* typification has been associated with area extension [46] and crop yield [47]. However, our results showed high variability in farm production. In this way, grouping them using this methodology was impossible. To the best of our knowledge, this study is the first to provide an agricultural and socioeconomic characterization of the *Musa* crop system in Huila, Colombia.

Crop variables differentiated Musa crops in Huila, whereas demographic variables showed little variation across farms and mainly described the overall system in the Department. It has been acknowledged that agricultural practice implementation and certification promote safe food consumption [60] and generate efficient production by farmers [78]. In general, agricultural practices have differentiated crop producers using typification [32–34]. The desire of farmers in the central and northern (Palermo) subregions for higher‐quality plantain products, as found in this study, provides access to new markets such as urban centers and international commerce, which is likely the connection between GAPc and the plant material correlation in the study. Colombia exports several agricultural commodities, including avocado [79], coffee [80], palm oil [81], and banana [82]. In all these products, crop certification is a requirement for selling agricultural products abroad. For socioeconomic variables, our results in terms of the gender of producers exhibit a trend similar to national data reported by the National Statistics Department of Colombia [83], which indicates that most producers engaged in agricultural activities in the country are male (71%). Decision‐making at the farm level is dominated by them with primary and high school education in Colombian rural areas [84], and the results about training ASOHOFRUCOL predominance align with studies in Colombia that highlight the relevance of technical trust networks between producers and technical assistants, who are key actors in disseminating technologies within the fruit chain [85]. Taken together, the cluster analysis (FAMD/HCPC) shows that agronomic practices were the primary elements differentiating Musa systems in Huila, which helps explain the contrasting levels of GAP adoption and phytosanitary conditions observed across the subregions.

GAPs and the main disease were the main variables that differentiated Musa crop Huila producers in each subregion. High variance in Musa quality and yield was observed in Group 1 (Pitalito and Timaná municipalities), generating a possible impact on local food security [86, 87]. In addition, the lack of crop sanitary practices in this cluster may be contributing to sanitary disorders, such as BED. In Colombia, BED is caused by the phytoplasma Candidatus asteris, which generates pseudostem swelling [88] and has been known since the 1980s [89]. Despite its long‐time presence, research on BED remains limited, even though it has economic importance in western Colombia [90]. Meanwhile, crop producers in Group 2, located in the central region (Garzón and Gigante), showed interest in Musa investment, including the use of external labor and implementation of GAPc. Despite this, soil and water analyses were not routinely performed and BED was the most relevant disease for this group. Similar results have shown that the lack of management in the crop system relates to high phytosanitary issues [91]. Time, dedication, and agricultural inputs must be invested in Musa cultivation in Groups 1 and 2, with a primary focus on sanitary limitations. On the contrary, Group 3 (Santa María) and Group 4 (Palermo) portrayed GAPc implementation and routinary soil and water analysis. These local practices in the north Huila Department presumably develop the highest crop yield production and the lowest variation in this parameter. In the eastern Colombian region, four typologies were identified based on crop management, productivity, and technology adoption [47]. These typologies, in turn, were linked to geographical localization, a finding consistent with the current research results. Overall, the cluster analysis highlights the importance of management quality and sanitary practices in Huila Musa production, establishing a clear connection to regional evidence on agricultural vulnerability and disease‐driven sustainability challenges.

Recent Latin America studies have highlighted how agricultural vulnerability (e.g., phytosanitary pressures) influences the sustainability of Musa production and demonstrated the value of combining typologies with epidemiological and spatial tools. In Colombia, susceptibility analyses by Rodríguez‐Yzquierdo et al. [92] indicated that extensive Musa areas remain highly exposed to *Fusarium oxysporum* f. sp. cubense Tropical Race 4 (Foc TR4), supporting regional warnings discovered by Martínez et al. [93]. These studies suggest that smallholder farms may be particularly susceptible to Foc TR4 due to limited monitoring capacity and disease‐control resources. Advances in disease prediction, such as random forest models for Black Sigatoka dynamics [94], offer tools to anticipate production losses and integrate sanitary risks into farm classification. Panama and Venezuela studies illustrate how multivariate methods improve the understanding of agricultural vulnerability and rural livelihoods. In Panama, research describes subsistence systems among indigenous groups in Bocas del Toro [95, 96], and applied economic studies in the Changuinola District show how structural poverty, diversification, and land use choices shape farming strategies [97]. In Venezuela, multivariate land suitability analyses have supported more accurate crop allocation [98]. These regional insights align with our findings in Huila, where differences in management practices and resource availability produced clear contrasts in productivity and agricultural pressures. This regional evidence reinforces the idea that local crop performance is deeply influenced by crop management and vulnerability conditions, which is especially evident in Huila’s intercropping between Musa and coffee systems.

Generally, coffee crops are cultivated together with Musa in the tropics [99]. In Africa, crops associated with banana have demonstrated that crop association contributes to the financial profitability of the cropping system, particularly in Benin [100], and banana‐coffee production is the most resilient in food security in Uganda [87]. In Colombia, coffee has been one of the most important agricultural commodities in the farm economy since the last century [101]. In the case of high pest and disease incidences in the Huila Department, it is likely connected to coffee because this cash crop is considered the main financial source for producers [102]. Therefore, smallholders can afford the maximum economic input and time investment in coffee [102], setting aside Musa crops. Furthermore, the frost that affected Brazil in 2021 [103] positively impacted Colombian coffee production, favoring producers and devaluing Musa plant management that year in the Huila Department. Thus, the Musa system in southwest Colombia is implemented as a secondary crop, with coffee retaining its status as the primary agricultural system, holding paramount importance for crop producers in this area [104, 105]. Plantains might be a potential crop to plant with coffee rather than bananas since the suitable areas in the Department for this last crop are very small. In this case, intercropping, i.e., cultivating two or more crops simultaneously on the same land, can be a powerful tool to enhance food security in the studied region, as well as in other developing countries [35, 87]. This farming technique offers numerous benefits that address various economic, environmental, and social challenges faced by smallholder farmers. The intercropping benefits boost biodiversity [35, 106], climate change resilience [25, 107, 108], and economic crop diversification [109, 110].

In this study, we demonstrated that sanitary and agricultural practices, rather than socioeconomic variables, are the main factors in Musa crop producers’ characterization, supporting our initial hypothesis. In each subregion of the Department, government authorities must pay attention to Musa producers, especially using the intercropping system plantain and coffee. Maintaining this association is essential because it ensures a balanced strategy in which the two crops complement each other [109], offering farmers diversified income sources and better labor management [110]. Despite receiving technical assistance, the evident deficiency in implementation of technologies—particularly in the central and southern subregions—appears to be linked to a shortage of financial resources when compared to the other subregions evaluated. Further studies on technology adoption are necessary to elucidate the status of farm practice applications in Huila. Finally, the possible south‐to‐north dissemination of BED observed in this study represents an important aspect that should be addressed in future research to clarify its epidemiological behavior in the Department.

### 4.1. Limitations of the Study

Climate variation might be related to large fluctuations in crop production and sanitary problems [86, 111]; however, this research did not consider climatic variables. Another issue was using the snowball sampling methodology for typology purposes. The reason was that there was a lack of official information about the total farms in Huila, and the focus of the project was on different farm associations (grouped by locality). Finally, even with the technical guidance provided by ASOHOFRUCOL professionals to the *Musa* producers, it is essential to concentrate the training on specific subjects such as disease and pest management. Technical and governmental support must be focused on each locality because there are different threats concerning crop practices in the four Huila subregions. For the *Musa* crops in this area, agroecological systems [112] might be considered an alternative to pest control.

## 5. Conclusions

This study provides the first typological assessment of *Musa* crop systems influenced by the phytosanitary status and the adoption of good agricultural practices across Huila subregions in Colombia. The results reveal spatial heterogeneity in crop management and disease pressures. Likewise, the close association between *Musa* and coffee highlights the intercropping potential to enhance diversification and local food security.

These findings provide valuable insights that may provide farm and governmental strategies to strengthen *Musa* production in the Huila Department. Future research should adopt probabilistic sampling, incorporate climatic and epidemiological risk data (e.g., BED and Sigatoka models), and apply socioeconomic resilience frameworks to strengthen typology‐based interventions. Overall, the study advances the understanding of smallholder Musaceae systems and offers a foundation for more sustainable, evidence‐based agricultural and policy strategies in tropical regions. These findings contribute to a comprehensive understanding of *Musa* crop management and socioeconomic factors in the southwest of Colombia and provide valuable guidance to crop producers and policymakers seeking to increase *Musa* productivity in an integrated manner.

## Conflicts of Interest

The authors declare no conflicts of interest.

## Funding

This study was funded by “Sistema General de Regalías de Colombia” Grant Number 2014 and “Ministerio de Ciencia, Tecnología e Innovación” (Ministry of Science, Technology and Innovation), with the project name “Desarrollo de modelo productivo de plátano con énfasis en material de propagación que atienda el problema de productividad y seguridad alimentaria derivadas de la emergencia económica, social y ecológica causada por el COVID‐19 en el departamento del Huila” (Development of a plantain production model with an emphasis on propagation material that addresses the productivity and food security issues arising from the economic, social, and ecological emergency caused by COVID‐19 in the Department of Huila) led by AGROSAVIA.

## Supporting Information

Table S1: The semistructured survey results for Musa crop systems in the southwestern region of Colombia.

## Supporting information


**Supporting Information** Additional supporting information can be found online in the Supporting Information section.

## Data Availability

All the data are available in the supporting information section.
