# Supplementary material for: Farm Typologies of Banana and Plantain Smallholders: Agricultural Practices and Disease Constraints in Department of Huila, Colombia
Source: Scientifica (Cairo). 2025 Dec 30;2025:3357641. doi: 10.1155/sci5/3357641 (PMC12782326; doi:10.1155/sci5/3357641)
Supplement: Supplementary file 1 — Supporting Information Additional supporting information can be found online in the Supporting Information section. [file SCI5-2025-3357641-s001.pdf]

Table S1. The semi-structured survey results for Musa crop systems in the southwestern region of Colombia.

| Farm | Producer's ag | Years of crop | CreditBank | Whatsapp_use | Technical training | Producer edu | Producer's ge | Internet use | Sewer system | WaterSource | WorkForce | Sub_region | Topography | Policulture Ma | Monoculture | Crop associat | Crop irrigation | MusaceaArea | PlantMaterial | GPA | MainDisease | Quality (%) | SoilAnal | WaterAnal | Wrap branch | RdtoAnual_ha | Pest management | Pest managem | Disease mana | Weed manage | trition manage | Plant pruning | Pseudostem elimination |
|---|---|---|---|---|---|---|---|---|---|---|---|---|---|---|---|---|---|---|---|---|---|---|---|---|---|---|---|---|---|---|---|---|---|
| 2 | 29-59 years ol | <10 years | CreditBank | Whatsapp usa | Asohofrucol t | Elemental sch | M | Internet | Not sewer sys | Torrent | FamExtraWo | Palermo | Steep Slope | 2 | Monoculture | Coffee crop | Not crop irrig | 3 | DominicHarto | GPA | Sigatoka | 90 | SoilAnal | WaterAnal | Wrap | 900 | Not pest management | Not pest mana | Cultural disea | Mechanical w | Chemical nutr | Plant pruning | Pseudostem elimination |
| 3 | ≥60 years old | <10 years | NoCreBank | Whatsapp usa | Asohofrucol t | High school | M | Internet | Not sewer sys | Torrent | FamExtraWo | Palermo | Steep Slope | 2 | Monoculture | Coffee crop | Not crop irrig | 3 | DominicHarto | GPA | Sigatoka | 90 | SoilAnal | WaterAnal | Wrap | 600 | Not pest management | Not pest mana | Cultural disea | Mechanical w | Chemical nutr | Plant pruning | Pseudostem elimination |
| 4 | ≥60 years old | <10 years | CreditBank | Whatsapp usa | Asohofrucol t | High school | M | Internet | Not sewer sys | Torrent | FamExtraWo | Palermo | Steep Slope | 2 | Monoculture | Coffee crop | Not crop irrig | 3 | DominicHarto | GPA | Sigatoka | 50 | SoilAnal | WaterAnal | Wrap | 2400 | Organic and cultural pest ma | ≥ 6 months | Cultural disea | Mechanical w | Chemical nutr | Plant pruning | Pseudostem elimination |
| 5 | 29-59 years ol | ≥30 years | CreditBank | Whatsapp usa | Asohofrucol t | Elemental sch | M | Internet | Not sewer sys | Torrent | FamExtraWo | Palermo | Steep Slope | 1.5 | No_Monocult | Coffee crop | Not crop irrig | 1,5 | DominicHarto | GPA | Sigatoka | 80 | SoilAnal | WaterAnal | Wrap | 1600 | Organic and cultural pest ma | ≥ 6 months | Cultural disea | Mechanical w | No chemical r | Plant pruning | Pseudostem elimination |
| 6 | 29-59 years ol | 10-30 years | CreditBank | Whatsapp usa | Asohofrucol t | High school | M | Internet | Not sewer sys | Torrent | FamExtraWo | Palermo | Steep Slope | 1.5 | Monoculture | Coffee crop | Not crop irrig | 2,3 | DominicHarto | GPA | Sigatoka | 100 | SoilAnal | WaterAnal | Wrap | 2000 | Organic and cultural pest ma | ≥ 6 months | Cultural disea | Mechanical w | Chemical nutr | Not plant prun | Pseudostem elimination |
| 7 | 29-59 years ol | 10-30 years | CreditBank | Whatsapp usa | Asohofrucol t | High school | M | Internet | Sewer System | Torrent | FamExtraWo | Palermo | Steep Slope | 0.5 | Monoculture | Coffee crop | Not crop irrig | 1 | DominicHarto | GPA | Sigatoka | 80 | SoilAnal | WaterAnal | Wrap | 2400 | Not pest management | Not pest mana | Cultural disea | Mechanical w | Chemical and | Plant pruning | Pseudostem elimination |
| 8 | 29-59 years ol | 10-30 years | CreditBank | Whatsapp usa | Asohofrucol t | High school | M | NoInternet | Sewer System | OtherWaterS | FamExtraWo | Palermo | Steep Slope | 2.5 | No_Monocult | Coffee crop | Not crop irrig | 2,5 | DominicHarto | GPA | Sigatoka | 73 | SoilAnal | WaterAnal | Wrap | 1000 | Chemical pest management | ≥ 6 months | Not disease m | Mechanical w | No chemical r | Plant pruning | Pseudostem elimination |
| 9 | 29-59 years ol | 10-30 years | CreditBank | Whatsapp usa | Asohofrucol t | Elemental sch | M | Internet | Sewer System | OtherWaterS | FamExtraWo | Palermo | Steep Slope | 4 | No_Monocult | Coffee crop | Not crop irrig | 4 | DominicHarto | GPA | Sigatoka | 90 | SoilAnal | WaterAnal | Wrap | 2083 | Not pest management | Not pest mana | Not disease m | Mechanical w | Chemical nutr | Plant pruning | Pseudostem elimination |
| 10 | ≥60 years old | ≥30 years | CreditBank | Whatsapp usa | Asohofrucol t | Elemental sch | M | Internet | Sewer System | OtherWaterS | FamExtraWo | Palermo | Steep Slope | 3 | No_Monocult | Coffee crop | Not crop irrig | 3 | DominicHarto | GPA | Sigatoka | 95 | SoilAnal | WaterAnal | Wrap | 2600 | Not pest management | Not pest mana | Not disease m | Mechanical ar | Chemical nutr | Plant pruning | Pseudostem elimination |
| 11 | 29-59 years ol | ≥30 years | CreditBank | Whatsapp usa | Asohofrucol t | Elemental sch | M | Internet | Sewer System | OtherWaterS | FamExtraWo | Palermo | Steep Slope | 3 | No_Monocult | Coffee crop | Not crop irrig | 3 | DominicHarto | GPA | Sigatoka | 100 | SoilAnal | WaterAnal | Wrap | 1200 | Chemical pest management | ≥ 6 months | Organic and c | Mechanical w | Chemical and | Plant pruning | Pseudostem elimination |
| 12 | 29-59 years ol | ≥30 years | NoCreBank | Whatsapp usa | Asohofrucol t | Elemental sch | M | Internet | Sewer System | OtherWaterS | FamExtraWo | Palermo | Steep Slope | 4 | No_Monocult | Coffee crop | Not crop irrig | 4 | DominicHarto | GPA | Sigatoka | 80 | SoilAnal | WaterAnal | Wrap | 2200 | Organic and cultural pest ma | ≥ 6 months | Cultural disea | Mechanical w | Chemical and | Plant pruning | Pseudostem elimination |
| 13 | ≥60 years old | ≥30 years | CreditBank | Whatsapp usa | Asohofrucol t | Elemental sch | M | Internet | Not sewer sys | Torrent | FamExtraWo | Palermo | Steep Slope | 1.5 | No_Monocult | Coffee crop | Crop irrigation | 1,5 | DominicHarto | GPA | Sigatoka | 80 | SoilAnal | WaterAnal | Wrap | 1200 | Organic and cultural pest ma | ≥ 6 months | Cultural disea | Mechanical w | Chemical nutr | Plant pruning | Pseudostem elimination |
| 14 | 29-59 years ol | ≥30 years | CreditBank | Whatsapp usa | Asohofrucol t | Elemental sch | M | NoInternet | Sewer System | OtherWaterS | FamExtraWo | Palermo | Steep Slope | 0.8 | Monoculture | Coffee crop | Not crop irrig | 1,8 | DominicHarto | GPA | Sigatoka | 60 | SoilAnal | WaterAnal | Wrap | 800 | Organic and cultural pest ma | ≥ 6 months | Not disease m | Mechanical w | Chemical nutr | Plant pruning | Pseudostem elimination |
| 15 | 29-59 years ol | 10-30 years | CreditBank | Whatsapp usa | Asohofrucol t | High school | M | Internet | Sewer System | OtherWaterS | FamExtraWo | Palermo | Steep Slope | 4 | Monoculture | Coffee crop | Not crop irrig | 7 | DominicHarto | GPA | Sigatoka | 100 | SoilAnal | WaterAnal | Wrap | 5200 | Not pest management | Not pest mana | Not disease m | Mechanical w | Chemical and | Plant pruning | Pseudostem elimination |
| 16 | ≥60 years old | ≥30 years | CreditBank | Whatsapp usa | Asohofrucol t | Elemental sch | M | NoInternet | Sewer System | Torrent | FamExtraWo | Palermo | Steep Slope | 2 | No_Monocult | Coffee crop | Not crop irrig | 2 | DominicHarto | GPA | Sigatoka | 100 | SoilAnal | WaterAnal | Wrap | 1600 | Chemical pest management | ≥ 6 months | Not disease m | Mechanical w | Chemical nutr | Plant pruning | Pseudostem elimination |
| 17 | 29-59 years ol | 10-30 years | CreditBank | Whatsapp usa | Asohofrucol t | Elemental sch | M | NoInternet | Not sewer sys | Torrent | ExtraWork | Santa_Maria | Steep Slope | 1.5 | No_Monocult | Coffee crop | Not crop irrig | 1,5 | DominicHarto | NoGPA | Sigatoka | 100 | NoSoilAnal | NotWaterAna | Wrap | 1200 | Organic and cultural pest ma | ≥ 6 months | Cultural disea | Mechanical w | Chemical nutr | Plant pruning | Pseudostem elimination |
| 18 | 29-59 years ol | 10-30 years | CreditBank | Whatsapp usa | Asohofrucol t | High school | M | NoInternet | Not sewer sys | Torrent | ExtraWork | Santa_Maria | Steep Slope | 3 | No_Monocult | Coffee crop | Not crop irrig | 3 | DominicHarto | NoGPA | Sigatoka | 100 | NoSoilAnal | NotWaterAna | Wrap | 1200 | Organic and cultural pest ma | ≥ 6 months | Cultural disea | Mechanical w | Chemical nutr | Plant pruning | Pseudostem elimination |
| 19 | 29-59 years ol | <10 years | NoCreBank | Whatsapp usa | Asohofrucol t | High school | M | NoInternet | Not sewer sys | Torrent | FamiliarWork | Santa_Maria | Steep Slope | 0 | Monoculture | Coffee crop | Not crop irrig | 2 | DominicHarto | NoGPA | Sigatoka | 100 | SoilAnal | NotWaterAna | Wrap | 1200 | Organic and cultural pest ma | ≥ 6 months | Not disease m | Mechanical w | Chemical nutr | Plant pruning | Pseudostem elimination |
| 20 | 29-59 years ol | 10-30 years | CreditBank | Whatsapp usa | Not technical | High school | M | NoInternet | Not sewer sys | Torrent | ExtraWork | Santa_Maria | Steep Slope | 2 | No_Monocult | Coffee crop | Not crop irrig | 2 | DominicHarto | NoGPA | Sigatoka | 100 | SoilAnal | NotWaterAna | NoWrap | 2000 | Not pest management | Not pest mana | Not disease m | Mechanical w | Chemical nutr | Plant pruning | Pseudostem elimination |
| 21 | 29-59 years ol | 10-30 years | CreditBank | Whatsapp usa | Asohofrucol t | High school | M | Internet | Not sewer sys | Torrent | ExtraWork | Santa_Maria | Steep Slope | 3 | No_Monocult | Coffee crop | Not crop irrig | 3 | DominicHarto | NoGPA | Sigatoka | 100 | NoSoilAnal | NotWaterAna | Wrap | 1000 | Not pest management | Not pest mana | Not disease m | Mechanical w | Chemical nutr | Plant pruning | Pseudostem elimination |
| 22 | 29-59 years ol | <10 years | CreditBank | Whatsapp usa | Asohofrucol t | Graduate/Bac | M | Internet | Not sewer sys | Torrent | ExtraWork | Santa_Maria | Steep Slope | 3 | No_Monocult | Coffee crop | Not crop irrig | 3 | DominicHarto | NoGPA | Sigatoka | 100 | NoSoilAnal | NotWaterAna | Wrap | 700 | Chemical pest management | ≥ 6 months | Not disease m | Mechanical w | Chemical nutr | Plant pruning | Pseudostem elimination |
| 23 | <29 years old | <10 years | CreditBank | Whatsapp usa | Asohofrucol t | High school | M | NoInternet | Not sewer sys | Torrent | ExtraWork | Santa_Maria | Steep Slope | 3 | No_Monocult | Coffee crop | Not crop irrig | 3 | DominicHarto | NoGPA | Sigatoka | 100 | SoilAnal | NotWaterAna | Wrap | 600 | Chemical pest management | ≥ 6 months | Cultural disea | Mechanical w | Chemical nutr | Plant pruning | Pseudostem elimination |
| 24 | 29-59 years ol | <10 years | CreditBank | Whatsapp usa | Asohofrucol t | High school | M | NoInternet | Not sewer sys | Torrent | ExtraWork | Santa_Maria | Steep Slope | 3 | No_Monocult | Coffee crop | Not crop irrig | 3 | DominicHarto | NoGPA | Sigatoka | 100 | NoSoilAnal | NotWaterAna | Wrap | 2000 | Chemical pest management | ≥ 6 months | Not disease m | Mechanical w | Chemical nutr | Plant pruning | Pseudostem elimination |
| 25 | 29-59 years ol | 10-30 years | CreditBank | Whatsapp usa | Asohofrucol t | High school | M | NoInternet | Not sewer sys | Torrent | ExtraWork | Santa_Maria | Steep Slope | 2.5 | No_Monocult | Coffee crop | Not crop irrig | 2,5 | DominicHarto | NoGPA | Sigatoka | 80 | SoilAnal | NotWaterAna | Wrap | 1000 | Not pest management | Not pest mana | Not disease m | Mechanical w | Chemical nutr | Plant pruning | Pseudostem elimination |
| 26 | 29-59 years ol | 10-30 years | CreditBank | Whatsapp usa | Asohofrucol t | High school | M | NoInternet | Not sewer sys | Torrent | FamiliarWork | Santa_Maria | Steep Slope | 1 | No_Monocult | Coffee crop | Not crop irrig | 1 | DominicHarto | NoGPA | Sigatoka | 100 | NoSoilAnal | NotWaterAna | Wrap | 1000 | Not pest management | Not pest mana | Not disease m | Mechanical ar | Chemical nutr | Plant pruning | Pseudostem elimination |
| 27 | <29 years old | <10 years | CreditBank | Whatsapp usa | Asohofrucol t | High school | M | Internet | Not sewer sys | Torrent | FamiliarWork | Santa_Maria | Steep Slope | 4 | Monoculture | Coffee crop | Crop irrigation | 7 | DominicHarto | NoGPA | Sigatoka | 80 | NoSoilAnal | NotWaterAna | Wrap | 600 | Organic and cultural pest ma | ≥ 6 months | Cultural disea | Mechanical ar | Chemical nutr | Plant pruning | Pseudostem elimination |
| 28 | 29-59 years ol | ≥30 years | CreditBank | Whatsapp usa | Not technical | Elemental sch | M | Internet | Not sewer sys | Torrent | FamiliarWork | Santa_Maria | Steep Slope | 1.5 | No_Monocult | Coffee crop | Not crop irrig | 1,5 | DominicHarto | NoGPA | Elephantiasis | 90 | SoilAnal | NotWaterAna | Wrap | 600 | Organic and cultural pest ma | ≥ 6 months | Cultural disea | Mechanical w | Chemical nutr | Plant pruning | Pseudostem elimination |
| 29 | ≥60 years old | 10-30 years | CreditBank | Not Whatsapp | Asohofrucol t | Elemental sch | M | NoInternet | Not sewer sys | Torrent | FamExtraWo | Santa_Maria | Moderate Slo | 2.5 | No_Monocult | Coffee crop | Not crop irrig | 2,5 | DominicHarto | NoGPA | Sigatoka | 90 | SoilAnal | NotWaterAna | Wrap | 400 | Not pest management | Not pest mana | Cultural disea | Mechanical ar | Chemical nutr | Plant pruning | Pseudostem elimination |
| 30 | 29-59 years ol | <10 years | CreditBank | Not Whatsapp | Asohofrucol t | Elemental sch | M | NoInternet | Not sewer sys | Torrent | FamExtraWo | Santa_Maria | Steep Slope | 0.1 | No_Monocult | Coffee crop | Not crop irrig | 0,1 | DominicHarto | NoGPA | Sigatoka | 95 | SoilAnal | NotWaterAna | Wrap | 200 | Not pest management | Not pest mana | Not disease m | Mechanical w | Chemical nutr | Plant pruning | Pseudostem elimination |
| 31 | ≥60 years old | 10-30 years | CreditBank | Not Whatsapp | Asohofrucol t | Elemental sch | M | NoInternet | Not sewer sys | Torrent | FamExtraWo | Santa_Maria | Steep Slope | 1 | No_Monocult | Coffee crop | Not crop irrig | 1 | DominicHarto | NoGPA | Sigatoka | 95 | NoSoilAnal | NotWaterAna | NoWrap | 2875 | Not pest management | Not pest mana | Cultural disea | Mechanical w | Chemical nutr | Plant pruning | Pseudostem elimination |
| 32 | 29-59 years ol | 10-30 years | NoCreBank | Whatsapp usa | Asohofrucol t | Elemental sch | M | NoInternet | Not sewer sys | Torrent | FamExtraWo | Santa_Maria | Steep Slope | 2 | No_Monocult | Coffee crop | Not crop irrig | 2 | DominicHarto | NoGPA | Elephantiasis | 95 | NoSoilAnal | NotWaterAna | Wrap | 2000 | Not pest management | Not pest mana | Not disease m | Mechanical w | Chemical nutr | Plant pruning | Pseudostem elimination |
| 33 | ≥60 years old | 10-30 years | CreditBank | Not Whatsapp | Not technical | Elemental sch | M | NoInternet | Not sewer sys | Torrent | FamExtraWo | Santa_Maria | Steep Slope | 3.5 | Monoculture | Coffee crop | Not crop irrig | 5 | DominicHarto | NoGPA | Sigatoka | 90 | NoSoilAnal | NotWaterAna | Wrap | 1800 | Organic and cultural pest ma | ≥ 6 months | Cultural disea | Mechanical ar | No chemical r | Plant pruning | Pseudostem elimination |
| 34 | ≥60 years old | <10 years | CreditBank | Not Whatsapp | Asohofrucol t | Elemental sch | M | NoInternet | Sewer System | OtherWaterS | FamExtraWo | Santa_Maria | Steep Slope | 1 | No_Monocult | Coffee crop | Not crop irrig | 1 | DominicHarto | NoGPA | Sigatoka | 95 | NoSoilAnal | NotWaterAna | Wrap | 450 | Not pest management | Not pest mana | Not disease m | Mechanical ar | No chemical r | Plant pruning | Pseudostem elimination |
| 35 | 29-59 years ol | <10 years | CreditBank | Whatsapp usa | Asohofrucol t | High school | M | Internet | Sewer System | OtherWaterS | FamExtraWo | Santa_Maria | Moderate Slo | 0 | Monoculture | Coffee crop a | Not crop irrig | 0,6 | DominicHarto | NoGPA | OtherDisease | 80 | NoSoilAnal | NotWaterAna | Wrap | 300 | Not pest management | Not pest mana | Not disease m | Mechanical w | Chemical nutr | Plant pruning | Pseudostem elimination |
| 36 | 29-59 years ol | 10-30 years | CreditBank | Whatsapp usa | Asohofrucol t | Elemental sch | M | Internet | Sewer System | OtherWaterS | FamExtraWo | Timana | Moderate Slo | 3 | No_Monocult | Coffee crop | Not crop irrig | 3 | GrossMichel | NoGPA | Elephantiasis | 80 | SoilAnal | NotWaterAna | NoWrap | 675 | Chemical pest management | ≥ 6 months | Not disease m | Mechanical w | Chemical nutr | Plant pruning | Pseudostem elimination |
| 37 | ≥60 years old | ≥30 years | CreditBank | Whatsapp usa | Asohofrucol t | High school | M | Internet | Sewer System | OtherWaterS | FamiliarWork | Timana | Moderate Slo | 2.5 | No_Monocult | Coffee crop | Not crop irrig | 2,5 | GrossMichel | NoGPA | Elephantiasis | 80 | NoSoilAnal | NotWaterAna | NoWrap | 200 | Organic and cultural pest ma | ≥ 6 months | Organic and c | Mechanical w | Chemical nutr | Plant pruning | Pseudostem elimination |
| 38 | ≥60 years old | ≥30 years | CreditBank | Not Whatsapp | Asohofrucol t | Elemental sch | F | NoInternet | Sewer System | OtherWaterS | FamiliarWork | Timana | Steep Slope | 2 | No_Monocult | Coffee crop | Not crop irrig | 2 | GrossMichel | NoGPA | Elephantiasis | 80 | NoSoilAnal | NotWaterAna | NoWrap | 935 | Chemical pest management | 1-6 months | Organic and chemical disease |  | No chemical r | Plant pruning | Pseudostem elimination |
| 39 | 29-59 years ol | 10-30 years | NoCreBank | Whatsapp usa | Not technical | High school | M | NoInternet | Sewer System | OtherWaterS | FamiliarWork | Timana | Moderate Slo | 3 | No_Monocult | Coffee crop | Not crop irrig | 3 | GrossMichel | NoGPA | Elephantiasis | 60 | NoSoilAnal | NotWaterAna | NoWrap | 1045 | Chemical pest management | 1-6 months | Not disease m | Mechanical ar | Chemical nutr | Plant pruning | Pseudostem elimination |
| 42 | 29-59 years ol | ≥30 years | NoCreBank |  | Not technical | Elemental sch | M | Internet | Sewer System | OtherWaterS | FamiliarWork | Timana | Moderate Slo | 3 | No_Monocult | Coffee crop | Not crop irrig | 3 | GrossMichel | NoGPA | Elephantiasis | 70 | NoSoilAnal | NotWaterAna | NoWrap | 105 | Chemical pest management | 1-6 months | Cultural disea | Mechanical w | Chemical nutr | Plant pruning | Pseudostem elimination |
| 43 | 29-59 years ol | 10-30 years | CreditBank | Not Whatsapp | Asohofrucol t | Elemental sch | M | Internet | Sewer System | OtherWaterS | FamiliarWork | Timana | Moderate Slo | 1 | No_Monocult | Coffee crop | Not crop irrig | 1 | GrossMichel | NoGPA | Elephantiasis | 0 | SoilAnal | NotWaterAna | Wrap | 375 | Organic and cultural pest ma | ≥ 6 months | Not disease m | Mechanical w | No chemical r | Plant pruning | Pseudostem elimination |
| 44 | 29-59 years ol | 10-30 years | NoCreBank | Whatsapp usa | Asohofrucol t | Elemental sch | M | NoInternet | Sewer System | OtherWaterS | FamiliarWork | Timana | Moderate Slo | 0.25 | No_Monocult | Coffee crop | Not crop irrig | 0,25 | GrossMichel | NoGPA | Elephantiasis | 90 | NoSoilAnal | NotWaterAna | NoWrap | 375 | Not pest management | Not pest mana | Organic and c | Mechanical w | Chemical nutr | Plant pruning | Pseudostem elimination |
| 45 | ≥60 years old | ≥30 years | NoCreBank | Whatsapp usa | Asohofrucol t | Elemental sch | M | NoInternet | Sewer System | OtherWaterS | FamiliarWork | Timana | Steep Slope | 0.75 | No_Monocult | Coffee crop a | Not crop irrig | 0,75 | GrossMichel | NoGPA | Elephantiasis | 80 | NoSoilAnal | NotWaterAna | NoWrap | 3000 | Not pest management | Not pest mana | Organic and c | Mechanical w | Chemical nutr | Plant pruning | Pseudostem elimination |
| 46 | 29-59 years ol | ≥30 years | NoCreBank | Not Whatsapp | Asohofrucol t | Elemental sch | M | Internet | Not sewer sys | OtherWaterS | ExtraWork | Timana | Moderate Slo | 0.05 | No_Monocult | Coffee crop | Not crop irrig | 0,05 | GrossMichel | NoGPA | Elephantiasis | 50 | NoSoilAnal | NotWaterAna | NoWrap | 740 | Not pest management | Not pest mana | Not disease m | Mechanical w | No chemical r | Plant pruning | Pseudostem elimination |
| 47 | <29 years old | 10-30 years | NoCreBank | Not Whatsapp | Asohofrucol t | Elemental sch | M | Internet | Sewer System | Torrent | FamExtraWo | Timana | Moderate Slo | 0.7 | No_Monocult | Coffee crop a | Not crop irrig | 0,7 | GrossMichel | NoGPA | Elephantiasis | 90 | NoSoilAnal | WaterAnal | Wrap | 454 | Not pest management | Not pest mana | Organic and c | Mechanical w | Chemical nutr | Plant pruning | Pseudostem elimination |
| 48 | 29-59 years ol | <10 years | CreditBank | Whatsapp usa | Asohofrucol t | Graduate/Bac | M | Internet | Sewer System | OtherWaterS | FamExtraWo | Timana | Moderate Slo | 0.02 | No_Monocult | Coffee crop | Not crop irrig | 0,02 | GrossMichel | NoGPA | Elephantiasis | 0 | SoilAnal | NotWaterAna | NoWrap | 2700 | Chemical pest management | ≥ 6 months | Organic and c | Mechanical w | Chemical nutr | Plant pruning | Pseudostem elimination |
| 49 | 29-59 years ol | ≥30 years | NoCreBank | Whatsapp usa | Asohofrucol t | Elemental sch | M | NoInternet | Sewer System | OtherWaterS | FamiliarWork | Timana | Steep Slope | 0.75 | No_Monocult | Coffee crop | Not crop irrig | 0,75 | GrossMichel | NoGPA | Elephantiasis | 80 | NoSoilAnal | NotWaterAna | Wrap | 3000 | Not pest management | Not pest mana | Organic and c | Mechanical w | Chemical nutr | Not plant prun | Pseudostem elimination |
| 50 | 29-59 years ol | ≥30 years | CreditBank | Not Whatsapp | Asohofrucol t | Elemental sch | F | Internet | Sewer System | OtherWaterS | FamExtraWo | Timana | Shallow Slope | 1 | No_Monocult | Coffee crop | Not crop irrig | 1 | GrossMichel | NoGPA | Elephantiasis | 0 | SoilAnal | NotWaterAna | Wrap | 2000 | Organic and cultural pest ma | ≥ 6 months | Organic and c | Mechanical w | Chemical nutr | Plant pruning | Pseudostem elimination |
| 53 | ≥60 years old | ≥30 years | CreditBank | Not Whatsapp | Not technical | Elemental sch | M | NoInternet | Sewer System | OtherWaterS | FamiliarWork | Timana | Moderate Slo | 0.3 | No_Monocult | Coffee crop | Not crop irrig | 0,3 | GrossMichel | NoGPA | Elephantiasis | 100 | NoSoilAnal | NotWaterAna | NoWrap | 5000 | Not pest management | Not pest mana | Not disease management |  | Chemical nutr | Plant pruning | Pseudostem elimination |
| 55 | ≥60 years old | 10-30 years | CreditBank | Whatsapp usa | Asohofrucol t | Elemental sch | M | NoInternet | Not sewer sys | Torrent | ExtraWork | Garzon | Steep Slope | 1.3 | No_Monocult | Coffee crop | Not crop irrig | 1,3 | DominicHarto | GPA | Elephantiasis | 90 | NoSoilAnal | NotWaterAna | Wrap | 1500 | Organic and cultural pest ma | 1-6 months | Organic and chemical disease |  | Chemical nutr | Plant pruning | Pseudostem elimination |
| 56 | ≥60 years old | 10-30 years | CreditBank | Whatsapp usa | Asohofrucol t | Elemental sch | M | NoInternet | Not sewer sys | Torrent | ExtraWork | Garzon | Shallow Slope | 4.5 | No_Monocult | Coffee crop | Not crop irrig | 4,5 | DominicHarto | GPA | Elephantiasis | 95 | SoilAnal | NotWaterAna | Wrap | 1500 | Not pest management | Not pest mana | Cultural disea | Mechanical w | Chemical nutr | Plant pruning | Pseudostem elimination |
| 57 | 29-59 years ol | <10 years | CreditBank | Not Whatsapp | Asohofrucol t | High school | F | Internet | Not sewer sys | Torrent | ExtraWork | Garzon | Shallow Slope | 1.5 | No_Monocult | Coffee crop | Not crop irrig | 1,5 | DominicHarto | GPA | Elephantiasis | 90 | NoSoilAnal | NotWaterAna | Wrap | 600 | Chemical pest management | 1-6 months | Organic and c | Mechanical w | Chemical nutr | Plant pruning | Pseudostem elimination |
| 58 | ≥60 years old | ≥30 years | CreditBank | Whatsapp usa | Asohofrucol t | Elemental sch | M | Internet | Sewer System | Torrent | ExtraWork | Garzon | Shallow Slope | 1 | No_Monocult | Coffee crop | Not crop irrig | 1 | DominicHarto | GPA | Elephantiasis | 90 | NoSoilAnal | NotWaterAna | Wrap | 750 | Not pest management | Not pest mana | Cultural disea | Mechanical w | Chemical nutr | Plant pruning | Pseudostem elimination |
| 59 | 29-59 years ol | 10-30 years | CreditBank | Whatsapp usa | Asohofrucol t | Elemental sch | M | Internet | Sewer System | OtherWaterS | ExtraWork | Garzon | Steep Slope | 3.5 | No_Monocult | Coffee crop | Not crop irrig | 3,5 | DominicHarto | GPA | Elephantiasis | 100 | NoSoilAnal | NotWaterAna | Wrap | 1500 | Chemical pest management | 1-6 months | Organic and chemical disease |  | Chemical and | Plant pruning | Pseudostem elimination |
| 60 | 29-59 years ol | <10 years | NoCreBank | Whatsapp usa | Asohofrucol t | Elemental sch | M | Internet | Sewer System | OtherWaterS | ExtraWork | Garzon | Steep Slope | 1.3 | No_Monocult | Coffee crop | Not crop irrig | 1,3 | DominicHarto | GPA | Elephantiasis | 90 | NoSoilAnal | NotWaterAna | Wrap | 1300 | Chemical pest management | ≥ 6 months | Organic and c | Mechanical w | Chemical nutr | Plant pruning | Pseudostem elimination |
| 61 | 29-59 years ol | 10-30 years | CreditBank | Whatsapp usa | Asohofrucol t | Graduate/Bac | F | NoInternet | Sewer System | OtherWaterS | FamExtraWo | Garzon | Moderate Slo | 1 | No_Monocult | Coffee crop | Not crop irrig | 1 | DominicHarto | GPA | Sigatoka | 90 | NoSoilAnal | NotWaterAna | Wrap | 1920 | Chemical pest management | 1-6 months | Organic and c | Mechanical w | Chemical nutr | Plant pruning | Pseudostem elimination |
| 62 | 29-59 years ol | <10 years | CreditBank | Whatsapp usa | Asohofrucol t | High school | M | Internet | Not sewer sys | OtherWaterS | FamiliarWork | Gigante | Shallow Slope | 1 | No_Monocult | Coffee crop | Not crop irrig | 1 | DominicHarto | GPA | OtherDisease | 80 | NoSoilAnal | NotWaterAna | Wrap | 880 | Chemical pest management | 1-6 months | Cultural disea | Mechanical w | Chemical nutr | Plant pruning | Pseudostem elimination |
| 63 | 29-59 years ol | <10 years | CreditBank | Whatsapp usa | Asohofrucol t | Elemental sch | M | Internet | Sewer System | Torrent | FamiliarWork | Gigante | Steep Slope | 0.5 | No_Monocult | Coffee crop | Crop irrigation | 0,5 | DominicHarto | GPA | Sigatoka | 95 | NoSoilAnal | NotWaterAna | Wrap | 2500 | Chemical pest management | 1-6 months | Cultural disea | Mechanical ar | Chemical nutr | Plant pruning | Pseudostem elimination |
| 64 | ≥60 years old | 10-30 years | CreditBank | Whatsapp usa | Asohofrucol t | Graduate/Bac | M | Internet | Sewer System | Torrent | FamExtraWo | Gigante | Moderate Slo | 1 | No_Monocult | Coffee crop | Not crop irrig | 1 | DominicHarto | GPA | Sigatoka | 100 | NoSoilAnal | NotWaterAna | Wrap | 1200 | Chemical pest management | ≥ 6 months | Organic and c | Mechanical ar | Chemical nutr | Plant pruning | Pseudostem elimination |
| 65 | ≥60 years old | <10 years | CreditBank | Whatsapp usa | Asohofrucol t | Graduate/Bac | M | Internet | Sewer System | Torrent | ExtraWork | Garzon | Moderate Slo | 0 | Monoculture | Coffee crop | Not crop irrig | 1,5 | DominicHarto | GPA | Sigatoka | 80 | NoSoilAnal | NotWaterAna | Wrap | 1800 | Not pest management | 1-6 months | Organic and chemical disease |  | Chemical nutr | Plant pruning | Pseudostem elimination |
| 66 | ≥60 years old | 10-30 years | CreditBank | Whatsapp usa | Asohofrucol t | Elemental sch | F | Internet | Sewer System | Torrent | ExtraWork | Garzon | Shallow Slope | 0.5 | No_Monocult | Coffee crop | Not crop irrig | 0,5 | DominicHarto | GPA | Elephantiasis | 90 | NoSoilAnal | NotWaterAna | Wrap | 750 | Organic and cultural pest ma | 1-6 months | Not disease m | Mechanical ar | Chemical nutr | Plant pruning | Pseudostem elimination |
| 67 | 29-59 years ol | <10 years | NoCreBank | Whatsapp usa | Asohofrucol t | Graduate/Bac | M | Internet | Sewer System | OtherWaterS | ExtraWork | Gigante | Moderate Slo | 2.5 | No_Monocult | Coffee crop | Not crop irrig | 2,5 | DominicHarto | GPA | Elephantiasis | 80 | NoSoilAnal | NotWaterAna | Wrap | 630 | Chemical pest management | ≥ 6 months | Organic and chemical disease |  | Chemical nutr | Plant pruning | Pseudostem elimination |
| 68 | ≥60 years old | 10-30 years | NoCreBank | Whatsapp usa | Asohofrucol t | Elemental sch | M | Internet | Sewer System | OtherWaterS | ExtraWork | Garzon | Moderate Slo | 1 | No_Monocult | Coffee crop | Not crop irrig | 1 | DominicHarto | GPA | Elephantiasis | 70 | NoSoilAnal | NotWaterAna | Wrap | 900 | Chemical pest management | 1-6 months | Cultural disea | Mechanical w | Chemical nutr | Plant pruning | Pseudostem elimination |
| 69 | 29-59 years ol | <10 years | CreditBank | Not Whatsapp | Asohofrucol t | High school | F | NoInternet | Sewer System | Torrent | FamExtraWo | Garzon | Steep Slope | 1.5 | No_Monocult | Not crop asso | Not crop irrig | 1,5 | DominicHarto | GPA | Sigatoka | 100 | NoSoilAnal | NotWaterAna | Wrap | 620 | Not pest management | Not pest mana | Not disease m | Mechanical ar | Chemical nutr | Plant pruning | Pseudostem elimination |
| 70 | ≥60 years old | 10-30 years | CreditBank | Whatsapp usa | Asohofrucol t | Elemental sch | M | Internet | Not sewer sys | Torrent | FamExtraWo | Garzon | Shallow Slope | 0.35 | No_Monocult | Coffee crop | Not crop irrig | 0,35 | DominicHarto | GPA | Elephantiasis | 95 | SoilAnal | NotWaterAna | Wrap | 600 | Not pest management | Not pest mana | Cultural disea | Mechanical w | Chemical nutr | Plant pruning | Pseudostem elimination |
| 71 | 29-59 years ol | ≥30 years | CreditBank | Not Whatsapp | Asohofrucol t | Elemental sch | M | Internet | Sewer System | Torrent | FamExtraWo | Garzon | Moderate Slo | 4 | No_Monocult | Coffee crop | Not crop irrig | 4 | DominicHarto | GPA | OtherDisease | 50 | NoSoilAnal | WaterAnal | Wrap | 1800 | Organic and cultural pest ma | 1-6 months | Cultural disea | Mechanical ar | Chemical nutr | Plant pruning | Pseudostem elimination |
| 72 | 29-59 years ol | ≥30 years | CreditBank | Whatsapp usa | Asohofrucol t | Elemental sch | M | Internet | Sewer System | OtherWaterS | FamExtraWo | Garzon | Moderate Slo | 1.38 | No_Monocult | Coffee crop | Not crop irrig | 1,38 | DominicHarto | GPA | Elephantiasis | 90 | NoSoilAnal | WaterAnal | Wrap | 450 | Not pest management | Not pest mana | Cultural disea | Mechanical w | Chemical nutr | Plant pruning | Pseudostem elimination |
| 73 | 29-59 years ol | 10-30 years | CreditBank | Whatsapp usa | Asohofrucol t | High school | F | Internet | Sewer System | Torrent | FamExtraWo | Gigante | Moderate Slo | 0.5 | No_Monocult | Coffee crop | Not crop irrig | 0,5 | DominicHarto | GPA | Sigatoka | 90 | SoilAnal | WaterAnal | Wrap | 180 | Organic and cultural pest ma | ≥ 6 months | Cultural disea | Mechanical w | Chemical and | Plant pruning | Pseudostem elimination |
| 74 | ≥60 years old | ≥30 years | NoCreBank | Whatsapp usa | Asohofrucol t | Elemental sch | M | Internet | Sewer System | OtherWaterS | FamExtraWo | Garzon | Moderate Slo | 1.9 | Monoculture | Coffee crop | Not crop irrig | 2,5 | DominicHarto | GPA | OtherDisease | 95 | NoSoilAnal | WaterAnal | NoWrap | 200 | Organic and cultural pest ma | ≥ 6 months | Cultural disea | Mechanical w | Chemical nutr | Plant pruning | Pseudostem elimination |
| 1 | ≥60 years old | 10-30 years | CreditBank | Whatsapp usa | Asohofrucol t | Elemental sch | M | Internet | Not sewer sys | Torrent | FamExtraWo | Palermo | Moderate Slo | 1 | Monoculture | Coffee crop | Crop irrigation | 2 | DominicHarto | GPA | Sigatoka | 40 | SoilAnal | WaterAnal | Wrap | 1200 | Organic and cultural pest ma | ≥ 6 months | Cultural disea | Mechanical w | Chemical and | Not plant prun | No pseudostem elimination |
| 40 | 29-59 years ol | ≥30 years | NoCreBank | Whatsapp usa | Asohofrucol t | Elemental sch | M | Internet | Sewer System | OtherWaterS | FamiliarWork | Timana | Steep Slope | 1.5 | No_Monocult | Coffee crop | Not crop irrig | 1,5 | GrossMichel | NoGPA | Elephantiasis | 70 | NoSoilAnal | NotWaterAna | NoWrap | 547 | Organic and cultural pest ma | 1-6 months | Not disease m | Mechanical w | Chemical and | Not plant prun | No pseudostem elimination |
| 51 | ≥60 years old | <10 years | NoCreBank | Whatsapp usa | Asohofrucol t | Graduate/Bac | M | NoInternet | Sewer System | OtherWaterS | FamExtraWo | Timana | Shallow Slope | 0.75 | No_Monocult | Coffee crop | Crop irrigation | 0,75 | DominicHarto | NoGPA | Elephantiasis | 0 | NoSoilAnal | NotWaterAna | NoWrap | 250 | Not pest management | Not pest mana | Not disease m | Mechanical ar | Chemical and | Not plant prun | No pseudostem elimination |
| 52 | ≥60 years old | ≥30 years | NoCreBank | Whatsapp usa | Asohofrucol t | Elemental sch | M | Internet | Sewer System | OtherWaterS | FamExtraWo | Timana | Moderate Slo | 1 | No_Monocult | Coffee crop | Not crop irrig | 1 | GrossMichel | NoGPA | Elephantiasis | 0 | NoSoilAnal | NotWaterAna | NoWrap | 600 | Chemical pest management | ≥ 6 months | Cultural disea | Mechanical w | Chemical nutr | Plant pruning | No pseudostem elimination |
| 54 | 29-59 years ol | 10-30 years | CreditBank | Not Whatsapp | Not technical | High school | M | NoInternet | Sewer System | OtherWaterS | FamiliarWork | Timana | Moderate Slo | 1.2 | No_Monocult | Coffee crop | Not crop irrig | 1,2 | GrossMichel | NoGPA | Elephantiasis | 100 | NoSoilAnal | NotWaterAna | NoWrap | 180 | Not pest management | Not pest mana | Not disease m | Mechanical w | Chemical nutr | Not plant prun | No pseudostem elimination |
| 75 | 29-59 years ol | ≥30 years | CreditBank | Whatsapp usa | Asohofrucol t | High school | M | Internet | Sewer System | OtherWaterS | FamExtraWo | Garzon | Moderate Slo | 1.5 | No_Monocult | Coffee crop | Not crop irrig | 1,5 | DominicHarto | GPA | Elephantiasis | 90 | NoSoilAnal | NotWaterAna | Wrap | 500 | Chemical pest management | ≥ 6 months | Cultural disea | Mechanical w | Chemical nutr | Not plant prun | No pseudostem elimination |

| Finca | Latitud (W) | Longitud | Altitud | PlantMaterial |
|---|---|---|---|---|
| 1 | 75°33'00701'' | 2°51'16.179'' | 1024 | DominicHarton |
| 2 | 75°34'36044'' | 2°51'57.540'' | 1400 | DominicHarton |
| 3 | 75°35'32171'' | 2°52'2.698'' | 1633 | DominicHarton |
| 4 | 75°35'28.941' | 2°52'18.142'' | 1643 | DominicHarton |
| 5 | 75°35'31.786' | 2°52'30273'' | 1645 | DominicHarton |
| 6 | 75°35'53.583' | 2°52'19.447'' | 1790 | DominicHarton |
| 7 | 75°35'55.849' | 2°52'17.663'' | 1828 | DominicHarton |
| 8 | 75°31'41.373' | 2°59'58.039'' | 1894 | DominicHarton |
| 9 | 75°31'30.053' | 2°59'33.745'' | 1871 | DominicHarton |
| 10 | 75°31'33.381' | 2°59'39.677'' | 1850 | DominicHarton |
| 11 | 75°32'04.714' | 2°58'50.341'' | 1840 | DominicHarton |
| 12 | 75°31'57.431' | 2°59'01.131'' | 1778 | DominicHarton |
| 13 | 75°32'15.886' | 2°59'22.878'' | 1759 | DominicHarton |
| 14 | 75°32'21.277' | 2°58'54.025''N | 1820 | DominicHarton |
| 15 | 75°31'39.077' | 2°59'36.418'' | 1775 | DominicHarton |
| 16 | 75°31'6.748'' | 3°0'016.353'' | 1865 | DominicHarton |
| 17 | 75°35'56.4'' | 2° 55’37.9’’ | 1493 | DominicHarton |
| 18 | 75°36'03.8'' | 2°55'31.9'' | 1527 | DominicHarton |
| 19 | 75°36'008.49 | 2°55'40.7'' | 1650 | DominicHarton |
| 20 | 75°37'22.016' | 2°53'55.380'' | 1853 | DominicHarton |
| 21 | 75°37'24,503' | 2°53'53,177'' | 1873 | DominicHarton |
| 22 | 75°36'07,418' | 2°55'34,022'' | 1557 | DominicHarton |
| 23 | 75°36'26,678' | 2°55'33,032'' | 1670 | DominicHarton |
| 24 | 75°36'21,186' | 2°55'28,016'' | 1540 | DominicHarton |
| 25 | 75°36'20,583' | 2°54'55,828' | 1668 | DominicHarton |
| 26 | 75°36'31,379' | 2°54'53,264'' | 1677 | DominicHarton |
| 27 | 75°36'13,702' | 2°54'44,592'' | 1617 | DominicHarton |
| 28 | 75°36'16,33'' | 2°54'56'' | 1573 | DominicHarton |
| 29 | 75°36'32,481' | 2°54'42,86'' | 1591 | DominicHarton |
| 30 | 75°34'57,783' | 2°55'22,579'' | 1803 | DominicHarton |
| 31 | 75°35'34,070' | 2°55'11,460'' | 1643 | DominicHarton |
| 32 | 75°35'20,78'' | 2°55'0092'' | 1825 | DominicHarton |
| 33 | 75°36'47,048' | 2°57'20,9968' | 1801 | DominicHarton |
| 34 | 75°36'48,68'' | 2°57'6,041'' | 1673 | DominicHarton |
| 35 | 75°38'14,96'' | 2°54'13,791'' | 1828 | DominicHarton |
| 36 | 75°55'27,631' | 1°57'8,114'' | 1200 | GrossMichel |
| 37 | 75°51'10,202' | 1°57'16,658'' | 1270 | GrossMichel |
| 38 | 75°53'17,661' | 1°57'58,594 | 1389 | GrossMichel |
| 39 | 75°55'26,884' | 1°57'10,937'' | 1190 | GrossMichel |
| 40 | 75°54'50,209' | 1°56'3,782'' | 1429 | GrossMichel |
| 42 | 75°53'24,931' | 1°58'15,066'' | 1355 | GrossMichel |
| 43 | 75°55'12,35'' | 1°57'05,951'' | 1243 | GrossMichel |
| 44 | 75°54'59,941' | 1°57'19,672'' | 1215 | GrossMichel |
| 45 | 75°54'55,829' | 1°56'31,872'' | 1380 | GrossMichel |
| 46 | 75°54'56,126' | 1°57'07,392'' | 1260 | GrossMichel |
| 47 | 75°52'38,266' | 1°58'32,708'' | 1171 | GrossMichel |
| 48 | 75°55'27,089' | 1°55'49,832'' | 1467 | GrossMichel |

| | | | | |
|---|---|---|---|---|
| 49 | 75°59'56,814' | 1°56'31,161'' | 1377 | GrossMichel |
| 50 | 75°55'13,302' | 1°56'58,431'' | 1259 | GrossMichel |
| 51 | 75°56'30,924' | 1°56'33,403'' | 1165 | DominicHarton |
| 52 | 75°53'38,463' | 1°58'53,920'' | 1363 | GrossMichel |
| 53 | 75°53'47,127' | 1°58'51,345'' | 1324 | GrossMichel |
| 54 | 75°56'19,698' | 1°56'19,772'' | 1238 | GrossMichel |
| 55 | 75°30'30,474' | 2°15'36,195'' | 1452 | DominicHarton |
| 56 | 75°30'30,678' | 2°15'31,581'' | 1417 | DominicHarton |
| 57 | 75°30'25,002' | 2°15'4,134'' | 1496 | DominicHarton |
| 58 | 75°38'29,149' | 2°15'40,915'' | 1437 | DominicHarton |
| 59 | 75°32'05,991' | 2°12'46,652'' | 1593 | DominicHarton |
| 60 | 75°32'00,173' | 2°12'44,474' | 1597 | DominicHarton |
| 61 | 75°31'39,893' | 2°13'41,442'' | 1500 | DominicHarton |
| 62 | 75°30'38,832' | 2°17'11,864'' | 1440 | DominicHarton |
| 63 | 75°31'5,289'' | 2°16'16,827'' | 1420 | DominicHarton |
| 64 | 75°32'35.785' | 2°15'24.991'' | 1324 | DominicHarton |
| 65 | 75°31'45.767' | 2°13'18.871'' | 1463 | DominicHarton |
| 66 | 75°31'16.218' | 2°14'52.997'' | 1332 | DominicHarton |
| 67 | 75°30'19,877' | 2°17'01,524'' | 1464 | DominicHarton |
| 68 | 75°32'27,494' | 2°13'20,771'' | 1452 | DominicHarton |
| 69 | 75°32'22,797' | 2°12'58,753'' | 1533 | DominicHarton |
| 70 | 75°32'03,332' | 2°13'58,620'' | 1438 | DominicHarton |
| 71 | 75°30'39,836' | 2°15'09,537'' | 1423 | DominicHarton |
| 72 | 75°30'17,771' | 2°15'00,110'' | 1685 | DominicHarton |
| 73 | 75°30'42,171' | 2°15'56,270'' | 1525 | DominicHarton |
| 74 | 75°31'29,094' | 2°15'07,319'' | 1323 | DominicHarton |
| 75 | 75°30'19,882' | 2°15'35,298'' | 1485 | DominicHarton |
